# Interactions between the *Aggregatibacter actinomycetemcomitans* secretin HofQ and host cytokines indicate a link between natural competence and interleukin-8 uptake

**DOI:** 10.1080/21505594.2018.1499378

**Published:** 2018-08-08

**Authors:** Tuuli Ahlstrand, Annamari Torittu, Heli Elovaara, Hannamari Välimaa, Marja T. Pöllänen, Sergo Kasvandik, Martin Högbom, Riikka Ihalin

**Affiliations:** a Department of Biochemistry, University of Turku, Turku, Finland; b Department of Virology, University of Helsinki, Helsinki, Finland; c Department of Oral and Maxillofacial Surgery, Helsinki University Hospital, Helsinki, Finland; d Institute of Dentistry, University of Turku, Turku, Finland; e Institute of Technology, University of Tartu, Tartu, Estonia; f Department of Biochemistry and Biophysics, Stockholm University, Stockholm, Sweden

**Keywords:** *Aggregatibacter actinomycetemcomitans*, periodontitis, bacterial outer membrane proteins, chemotactic cytokines, interleukin-8, protein interaction domains and motifs, DNA-binding proteins

## Abstract

Naturally competent bacteria acquire DNA from their surroundings to survive in nutrient-poor environments and incorporate DNA into their genomes as new genes for improved survival. The secretin HofQ from the oral pathogen *Aggregatibacter actinomycetemcomitans* has been associated with DNA uptake. Cytokine sequestering is a potential virulence mechanism in various bacteria and may modulate both host defense and bacterial physiology. The objective of this study was to elucidate a possible connection between natural competence and cytokine uptake in *A. actinomycetemcomitans*. The extramembranous domain of HofQ (emHofQ) was shown to interact with various cytokines, of which IL-8 exhibited the strongest interaction. The dissociation constant between emHofQ and IL-8 was 43 nM in static settings and 2.4 μM in dynamic settings. The moderate binding affinity is consistent with the hypothesis that emHofQ recognizes cytokines before transporting them into the cells. The interaction site was identified via crosslinking and mutational analysis. By structural comparison, relateda type I KH domain with a similar interaction site was detected in the *Neisseria meningitidis* secretin PilQ, which has been shown to participate in IL-8 uptake. Deletion of *hofQ* from the *A. actinomycetemcomitans* genome decreased the overall biofilm formation of this organism, abolished the response to cytokines, *i.e*., decreased eDNA levels in the presence of cytokines, and increased the susceptibility of the biofilm to tested β-lactams. Moreover, we showed that recombinant IL-8 interacted with DNA. These results can be used in further studies on the specific role of cytokine uptake in bacterial virulence without interfering with natural-competence-related DNA uptake.

## Introduction

*Aggregatibacter actinomycetemcomitans* is a gram-negative, opportunistic, oral pathogen that is associated with both aggressive and chronic forms of periodontitis [1–3]. In periodontitis, an intensified host inflammatory response against subgingival biofilms, consisting primarily of inflammophilic gram-negative species, causes the destruction of gingival tissue and alveolar bone (for reviews see refs. [4,5]) In this excessive inflammation neutrophils produce disproportionate amounts of reactive oxygen species (ROS) that take part in the tissue destruction (for review, see ref. [6]). In the most severe cases of periodontitis, extensive destruction of tooth-supporting tissues leads to tooth detachment [7]. In bacterial biofilm communities, such as subgingival biofilms, bacteria are surrounded by protective extracellular matrices (EMs), which are currently under intensive investigation, and novel ways of treating biofilm infections are being developed (for a review, see ref. [8]). EMs consist of proteins, extracellular DNA (eDNA) and polysaccharides, such as poly-N-acetylglucosamine (PGA), which is the major polysaccharide in *A. actinomycetemcomitans* biofilms [9]. However, *A. actinomycetemcomitans* requires eDNA for cohesive biofilm formation, efficient adhesion to surfaces [10,11], and leukotoxin attachment to the bacterial membrane [12].

In addition to forming a protective biofilm, *A. actinomycetemcomitans* has many virulence factors, including secreted toxins, such as leukotoxin and cytolethal distending toxin (for reviews, see refs [13,14].), which harm host tissues and defense systems. We have recently discovered a new type of virulence mechanism in *A. actinomycetemcomitans*; the biofilm of this bacterium sequesters the human inflammatory cytokines and chemokines interleukin (IL)-1β, IL-8 and IL-6 and transfers these molecules into the cells [15–17]. This cytokine uptake changes the EM composition of the biofilm [17] and decreases the metabolic activity of the biofilm [15], which may increase the resilience of bacteria in hostile environments. As the bacteria internalize cytokines, the cytokine concentrations change locally, which may further alter human leukocyte activity at the site.

Recently, we discovered an outer membrane lipoprotein named bacterial IL receptor I (BilRI) in *A. actinomycetemcomitans*; this lipoprotein binds IL-1β and other cytokines, such as IL-8, IL-10, and tumor necrosis factor (TNF)-α [17,18]. The binding affinity of BilRI to cytokines is rather low; therefore, BilRI may act by concentrating cytokines on cell membranes, after which the cytokines are transferred to the next component in the cytokine uptake machinery. However, the machinery via which the cytokines enter *A. actinomycetemcomitans* cells has yet to be identified.

*Neisseria meningitidis* has a pore-forming outer membrane secretin, PilQ (NmPilQ), which is involved in the production of type IV pili and binds IL-8 and TNF-α[19]. *A. actinomycetemcomitans* possesses a NmPilQ homolog, the secretin HofQ, which forms a channel through the outer membrane. We named the protein HofQ due to the homology of this protein with the *Escherichia coli* secretin HofQ [20]; however, this protein is also known as ComE because it is located downstream of the *comABCD* locus in *A. actinomycetemcomitans* [21]. This locus is involved in the uptake of eDNA by this bacterium, a characteristic known as natural competence [21]. We have previously solved the three-dimensional structure of the extramembranous domains of HofQ (emHofQ) and demonstrated the direct binding of emHofQ to double-stranded DNA [20]. However, since NmPilQ is involved in the uptake of IL-8 and TNF-α, the aim of this study was to elucidate the possible cytokine-binding role of HofQ.

Our results showed that emHofQ bound various cytokines, and the strongest affinity was observed for IL-8. A domain homologous to the emHofQ domain that interacts with IL-8 is present in NmPilQ. Deletion of the *hofQ* gene from *A. actinomycetemcomitans* abolished the biofilm response to cytokines IL-1β and IL-8, *i.e*., the decrease in eDNA levels in the presence of cytokines. Moreover, we showed that recombinant IL-8 interacted with DNA. Thus, the secretin HofQ might be a channel through which IL-8 is taken up by *A. actinomycetemcomitans* cells in a process that combines the uptake of DNA with the uptake of cytokines.

## Results

### Recombinant emHofQ bound various cytokines

**Figure 1.**
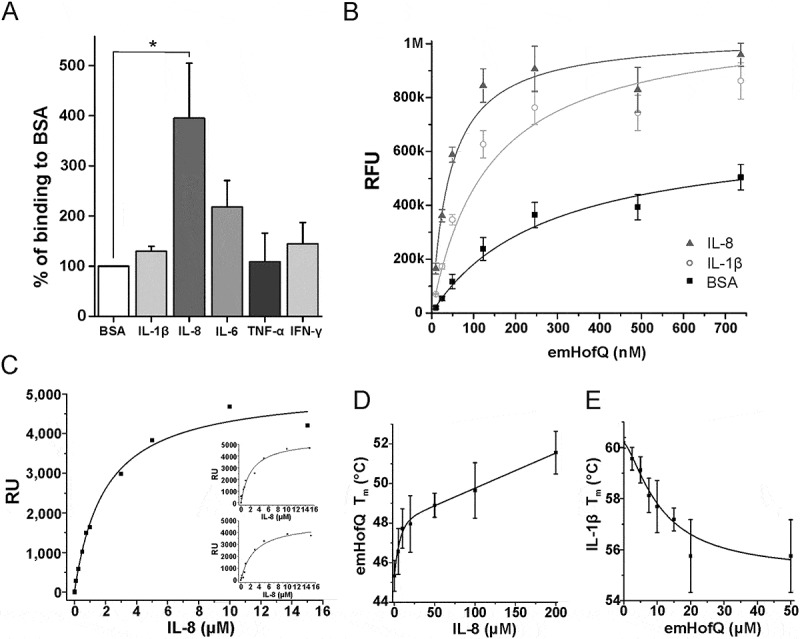
emHofQ Interacts with Cytokines. (A) Binding of cytokines by emHofQ was determined with ELISA. Data are presented as percentage of binding to control protein BSA. Binding of emHofQ to IL-8 was significantly higher than that to BSA (p-value 0.029, Mann-Whitney U-test); N = 4. (B) Interaction with IL-8 and IL-1β was measured with varying emHofQ concentrations using TRFIA. Dissociation constants were calculated for the binding curves: 43 ± 4 nM (emHofQ-IL-8) and 140 ± 20 nM (emHofQ-IL-1β). Data were obtained from three independent experiments and are presented as the mean± SD. BSA was used as a negative binding control. (C) Binding of IL-8 to immobilized emHofQ at different IL-8 concentrations was determined by surface plasmon resonance. The dissociation constant was estimated from the binding curves as being 2.4 ± 1.3 μM. Three independent experiments were performed; one representative image is shown as the primary figure, and the two other images are shown as insets. (D) In the thermal shift assay, the melting temperature (T_m_) of emHofQ (45.3°C) increased when emHofQ and IL-8 were co-incubated. At the highest tested IL-8 concentration of 200 μM, the T_m_ obtained was 51.7°C; N = 4–6. (E) In contrast, the T_m_ of IL-1β (60.2°C) decreased when IL-1β was incubated with emHofQ. With 50 μM emHofQ, the T_m_ obtained was 55.8°C; N = 3–4.

First, the steady-state binding of His-tagged emHofQ to an array of immobilized cytokines was investigated with a microplate assay. Recombinant emHofQ interacted with IL-8, IL-6 and IL-1β, of which the interaction with IL-8 was the most efficient (p = 0.029, Mann-Whitney U-test, Figure 1(A)): emHofQ bound to IL-8 4.0 times better than to the control (BSA), while the binding of emHofQ to IL-1β was 1.3 times better than that to the control.

### Binding of emHofQ to IL-8 was stronger than that to IL-1β

To obtain quantitative information on the binding affinities of emHofQ to IL-8 (strong binding) and IL-1β (weak binding), we then performed a time-resolved fluorometric immunoassay (TRFIA). In this assay, the cytokine amounts in the microtiter wells were kept constant, and the amount of emHofQ was varied (10–740 nM). We then used one-to-one fitting models to calculate the dissociation constants (K_d_) for the binding curves (Figure 1(B)). The calculated K_d_ values for the emHofQ-IL-8 and emHofQ-IL-1β interactions were 43 ± 4 nM and 140 ± 20 nM, respectively.

### Immobilized emHofQ bound IL-8 with moderate affinity

We examined the binding of recombinant mature IL-8 to immobilized emHofQ by surface plasmon resonance (SPR). The binding of IL-8 was concentration dependent and therefore, by definition, specific (Figure 1(C)). At concentrations lower than 1 μM, equilibrium was reached during the injection, but at higher concentrations, which were necessary for the determination of K_d_, the equilibrium was not reached, presumably due to high unspecific binding of IL-8 to both the reference and emHofQ channels. This behavior was reproducible. The fit of the data suggested one-to-one binding, and the apparent binding constant was moderate (K_d_(*app*) = 2.4 ± 1.3 μM).

IL-1β bound reproducibly (N = 2) to emHofQ, but the affinity was much lower than that of IL-8. All the data points obtained (up to 50 μM) were in the linear part of the binding curve, and we were not able to determine the binding constant for the interaction (data not shown).

### IL-8 stabilized the structure of emHofQ, while emHofQ destabilized the structure of IL-1β

A thermal shift assay was conducted to determine whether the interaction of emHofQ with IL-8 or IL-1β changes the thermal denaturation temperatures of the interacting proteins. Cytokines were co-incubated with varying emHofQ concentrations and supplemented with the SYPRO orange fluorescent label, which binds all hydrophobic surfaces. During the experiment, increasing temperatures further expose the hydrophobic regions of the proteins, thereby elevating the fluorescent signal. The melting temperature (T_m_) of the protein is estimated from the melting curve obtained from the fluorescent signal. The T_m_ of emHofQ increased from 45.3°C to 51.7°C upon co-incubation with 200 μM IL-8 (Figure 1(D)); therefore, the interaction with IL-8 had a stabilizing effect on the emHofQ fold.

When emHofQ was co-incubated with IL-1β, only the T_m_ of IL-1β was obtainable from the melting curves (data not shown). Therefore, the experiment was performed with a constant IL-1β concentration and by varying the emHofQ concentration. The T_m_ of IL-1β decreased from 60.2°C to 55.8°C upon co-incubation with 50 μM emHofQ (Figure 1(E)). Thus, the binding of emHofQ to IL-1β destabilizes the IL-1β fold.

### Crosslinking of emHofQ with IL-8

**Figure 2.**
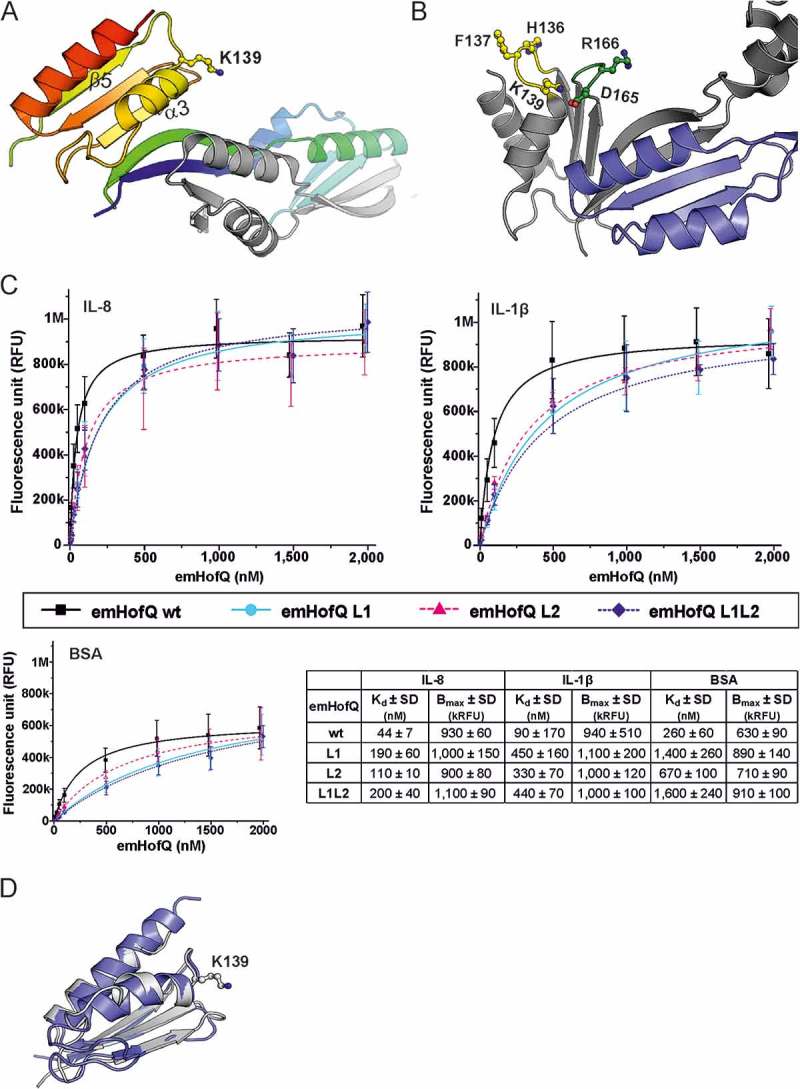
The type I KH domain of emHofQ interacted with IL-8. (A) Homodimeric structure of emHofQ (PDB: 2Y3M). Monomer A is colored blue-red from the N terminus to the C terminus, and monomer B is in gray. K139, which involved in the IL-8 interaction, and as the secondary structure elements β5 and α3, which are located next to the interacting loop, are indicated. (B) A total of five mutations were made in two loops (L1 and L2) in the type I KH domain of emHofQ: L1 (H136A, F137A, K139A) and L2 (D165A, R166A). (C) Mutated emHofQ variants bound to IL-8 and IL-1β with lower affinity than the wild-type emHofQ. BSA was used as a negative binding control (smaller graph). Data were obtained from three independent experiments and are presented as the mean± SD. Dissociation constants (K_d_) and values of maximum binding (B_max_) for each variant are listed in the table. (D) Structural comparison of the C-terminal type I KH domain of emHofQ (residues 126–191, in gray) with the N1-domain of NmPilQ (residues 439–516 (PDB id: 4AV2), in blue).

The crosslinking experiment links lysine residues in interacting proteins that are in close proximity to each other. The co-incubation of recombinant IL-8 and emHofQ with a crosslinking agent produced an additional band of approximately 30 kDa on an SDS-PAGE gel. Mass spectrometric analysis of the crosslinked protein band revealed that the sites surrounding Lys15 in IL-8 and Lys139 in the C-terminal domain of emHofQ are in close proximity in the interaction complex (Figure 2(A)). However, the binding of emHofQ to IL-1β was too weak, and we could not get any information from the crosslinking of this ligand (data not shown).

### Two loop regions in the type I KH domain of emHofQ contribute to the binding of IL-8 and IL-1β

To further characterize the IL-8 interaction site of emHofQ, we mutated large residues in the proposed interaction site of emHofQ. Three mutations (H136A, F137A and K139A) were made in the loop (between β5 and α3, hereafter referred to as L1) that was indicated as being close to the interaction site by the crosslinking analysis (Figure 2(C)). Two mutations (D165A and R166A) were introduced in a loop (loop 2, L2) that was located in the same plane of the protein as L1 (Figure 2(C)). The third mutant was a combination of all five abovementioned mutations (L1L2). The mutations decreased the binding affinity of emHofQ to IL-8 by two- to fourfold (Figure 2(D)), which indicates that these loops play a role in the interaction. The apparent K_d_ values for the wild-type emHofQ and the emHofQ mutants L1, L2 and L1L2 were 44 ± 7 nM, 190 ± 60 nM, 110 ± 20 nM and 200 ± 40 nM, respectively. The K_d_ values were calculated from the binding curves obtained from the TRFIA.

We next examined the binding of the emHofQ mutant proteins to IL-1β, and the mutations also decreased the binding affinity in this interaction, by four to fivefold (Figure 2(D)). The obtained K_d_ values for wild-type emHofQ and the emHofQ mutants L1, L2 and L1L2 were 90 ± 170 nM, 450 ± 160 nM, 330 ± 70 nM and 440 ± 70 nM, respectively. These results indicate that the IL-1β-emHofQ interaction occurs close to the IL-8-emHofQ interaction site. Overall, the mutations in loop 1 seem to have the greatest effect on the binding affinity.

### The IL-8-binding C-terminal domain of emHofQ shares homology with the N1 domain of NmPilQ

The crosslinking experiments indicated that Lys139 in the C-terminal domain of emHofQ is involved in the interaction with IL-8. In a previous study, we have shown that the C-terminal domain of emHofQ possesses a type I KH domain fold [20]. Lys139 is located in the exposed loop linking the β5 and α3 secondary structure elements of the KH domain (Figure 2(A)). Notably, another protein that has been shown to be involved in cytokine uptake in bacteria is the type IV pilus secretin NmPilQ. While the cytokine-binding region of NmPilQ has not been identified, notably, the N1 domain of this protein shares sequence homology with the C-terminal domain of emHofQ (26% sequence identity). This level of sequence similarity is a strong indication of a shared fold. A recent study of the type IV pili trans-periplasmic channel in *N. meningitidis* produced a structural model of the NmPilQ protein based on combined NMR, EM and CS-ROSETTA homology modeling [22]. In this structure (PDB id: 4AV2), the N1 domain adopts the same fold as the C-terminal domain of emHofQ (Figure 2(B)).

### IL-8 binds to linear DNA

**Figure 3.**
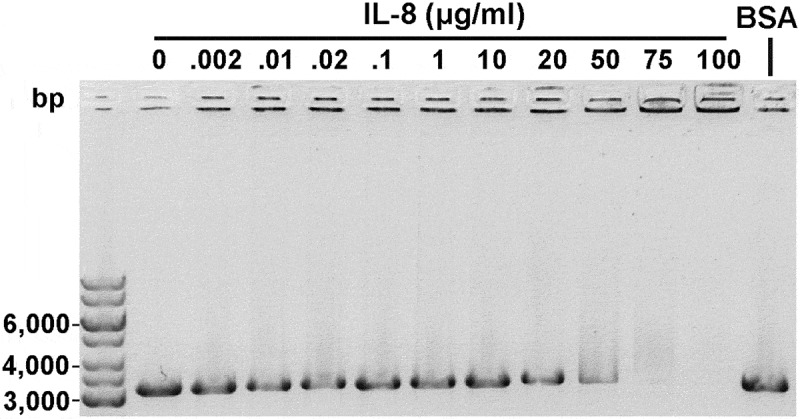
Recombinant IL-8 interacted with linear dsDNA. Linear *A. actinomycetemcomitans* USS DNA (31 μg/ml) was incubated with varying amounts of IL-8 for 30 min at RT and run on a 0.8% agarose gel (EMSA). Interaction between the DNA and IL-8 was observed with high IL-8 amounts as the formed complexes were trapped in the wells. BSA (10 mg/ml) was used as a negative control.

In previous studies, HofQ was shown to play a role in DNA binding [20]. Moreover, a loss of function mutation in the *hofQ*/*comE* gene was linked to a loss of competence [21]. Electrophoretic mobility shift assay (EMSA) was used to examine whether IL-8 interacts with linear DNA containing the *A. actinomycetemcomitans* uptake signal sequence (USS). When the DNA (31 μg/ml) was incubated with increasing amounts of IL-8, DNA-IL-8 complexes were observed after 50 μg/ml IL-8; the DNA remained in the wells and were unable to move through the agarose gel (Figure 3). A similar interaction was not observed using IL-1β (data not shown).

### The HofQ deletion mutant exhibited altered biofilm formation

**Figure 4.**
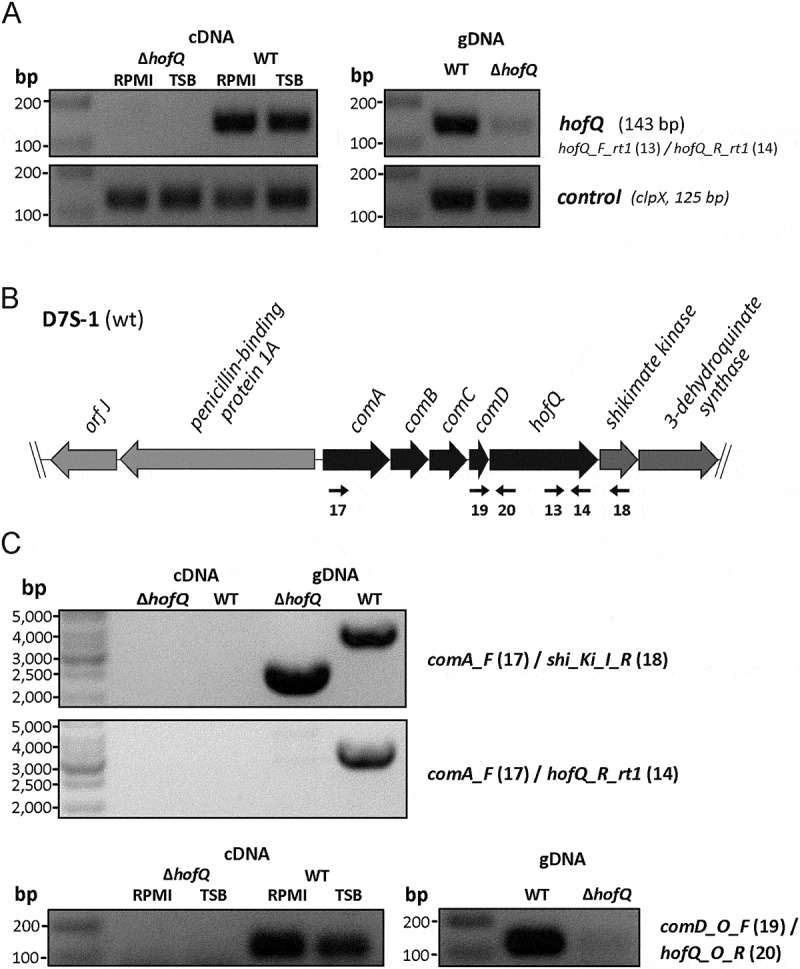
Verification of the Δ*hofQ* mutant and expression of *hofQ* from the *com* locus. (A) Deletion of the *hofQ* gene from *A. actinomycetemcomitans* D7S was verified by extracting the genomic DNA and performing PCR with flanking primers. The deletion was further verified at the transcriptional level by extracting RNA and performing reverse transcription -PCR. No *hofQ* gDNA or cDNA was observed in the *A. actinomycetemcomitans ΔhofQ* mutant. In addition, in the D7S wild-type strain, *hofQ* was transcribed at a higher level in minimal (RPMI) medium than in rich (TSB) culture medium. The control gene *clpX* was expressed and transcribed at the same level in both strains and culture conditions. (B) Genetic organization of the *com* locus in *A. actinomycetemcomitans* D7S. DNA in the *com* locus is marked with dark arrows, and additional ORFs outside the *com* locus are noted with gray arrows. Small black arrows indicate the locations of the primers (numbered) used for operon characterization. (C) Neither the gene locus from *comA* to *shiKi* nor that from *comA* to *hofQ* was detected as a single transcription unit, even in wild-type D7S, indicating that these genes are organized in different operons. However, the *comD* and *hofQ* genes were co-transcribed in wild-type D7S but were absent in the *ΔhofQ* strain. In wild-type *A. actinomycetemcomitans*, the *comD-hofQ* operon was transcribed more in minimal culture medium (RPMI) than in rich culture medium. Primers used for operon characterization are indicated by their names and numbers (listed in Table 4) next to each panel.

To elucidate the role of HofQ in the phenotype of *A. actinomycetemcomitans* we deleted the *hofQ* gene from the genome by Cre-*loxP*-mediated recombination [23,24]. Successful deletion of the *hofQ* gene was confirmed at the genomic and transcriptional levels. In the wild-type *A. actinomycetemcomitans* D7S strain, *hofQ* was transcribed more efficiently in minimal medium than in rich medium (Figure 4(A)). Although an operon prediction database (Database of prOkaryotic OpeRons) suggested that *hofQ* is part of an operon with the gene cluster *comABCD* (Figure 4(B)), *hofQ* was actually co-transcribed with only *comD*, which is located upstream of and adjacent to *hofQ* (Figure 4(C)).

In the deletion strain, biofilm formation decreased (Table 1). The total biofilm mass of this strain was half of that of the wild-type strain (p = 0.029; Mann-Whitney U-test), and the amounts of all of the major biofilm components (protein, eDNA, PGA) decreased significantly (with p-values of 0.029, 0.029, and 0.029, respectively; Mann-Whitney U-test). Incubation with 10 ng/ml IL-8 or IL-1β for 22 h did not alter the biofilm composition of the *A. actinomycetemcomitans* Δ*hofQ* strain (Table 2). In wild-type *A. actinomycetemcomitans*, IL-1β increased the PGA levels (p = 0.029; Mann-Whitney U-test) and decreased the total protein amount (p = 0.029; Mann-Whitney U-test), while IL-8 slightly decreased the biofilm mass (p = 0.029; Mann-Whitney U-test). Furthermore, incubation with IL-8 or IL-1β significantly reduced the amount of eDNA (with p-values of 0.029 and 0.029, respectively; Mann-Whitney U-test) in wild-type *A. actinomycetemcomitans* (Table 2).

### Deletion of hofQ increased the susceptibility of A. actinomycetemcomitans to ampicillin and amoxicillin/clavulanic acid

**Figure 5.**
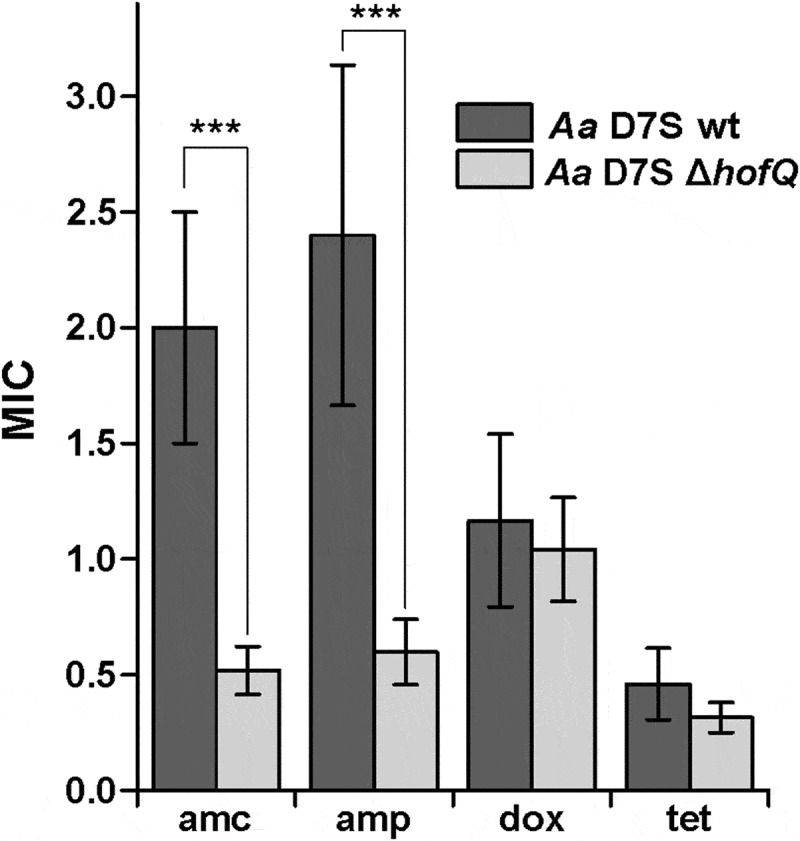
Deletion of *hofQ* increased the antimicrobial susceptibility of *A. actinomycetemcomitans* to β-lactams. Deletion of the *hofQ* gene made *A. actinomycetemcomitans* more susceptible to ampicillin (amp, p = 0.001) and amoxicillin/clavulanic acid (amc, p = 0.001) but did not significantly alter the susceptibility to tetracycline (tet, p = 0.093) or doxycycline (dox, p = 0.485). MIC values were determined from the scale on the Etest® strip after 45–48 h of incubation. Data were obtained from 5–9 independent experiments and are presented as the mean± SD.

Next, we investigated whether deletion of *hofQ* would affect the antimicrobial resistance of *A. actinomycetemcomitans*. When a panel of four antimicrobials were tested, wild-type *A. actinomycetemcomitans* was observed to be susceptible to amoxicillin/clavulanic acid, doxycycline and tetracycline while exhibiting intermediate resistance to ampicillin (Clinical and Laboratory Standards Institute (CLSI, Wayne, PA, USA) MIC criteria). Deletion of *hofQ* increased the susceptibility of *A. actinomycetemcomitans* to ampicillin (p = 0.001, Mann-Whitney U-test) and amoxicillin/clavulanic acid (p = 0.001, Mann-Whitney U-test), while the susceptibility to doxycycline and tetracycline was not altered (p-values of 0.485 and 0.093, respectively; Mann-Whitney U-test) (Figure 5).

### ΔhofQ mutants induced leukocyte ROS production similarly to wild-type cells

**Figure 6.**
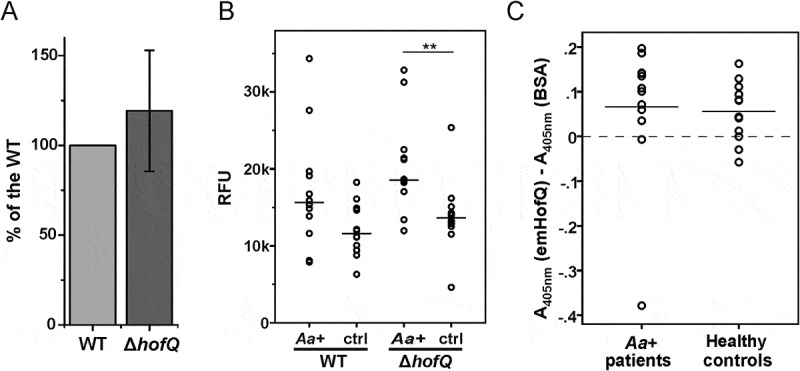
emHofQ was not antigenic in *A. actinomycetemcomitans-*positive periodontitis patients, and HofQ did not enhance the production of ROS by leukocytes when stimulated with *A. actinomycetemcomitans* cells. (A) Compared with the wild-type strain, the *hofQ* deletion did not significantly affect the production of ROS by leukocytes (p = 0.442). ROS production was measured as chemiluminescence, and the peak values obtained for the wild-type and Δ*hofQ* mutant *A. actinomycetemcomitans* strains were compared. Bacteria were pretreated with plasma collected from a healthy individual, and leukocytes were freshly isolated from the plasma of healthy control subjects (N = 8). (B) Opsonization efficiency of the sera collected from *A. actinomycetemcomitans*-positive patients was higher than that of the healthy control sera, but these sera significantly only enhanced the production of ROS by leukocytes when the Δ*hofQ* mutant was used (p = 0.005). Peak values of the chemiluminescence reactions are reported for 12 patient sera and 12 control sera. The horizontal lines indicate the mean values of each sample set; N = 1–3. (C) Sera collected from *A. actinomycetemcomitans*-positive patients did not have higher emHofQ antibody levels than sera from healthy control subjects when tested with ELISA. A total of 12 patient sera and 12 control sera were tested. Data corresponding to nonspecific binding to the negative control (BSA) was subtracted from the data obtained for emHofQ binding.

Because HofQ is located in the outer membrane of *A. actinomycetemcomitans*; thus, this protein might play a role in the recognition of this bacterium by host immune cells, which then induces the production of ROS by leukocytes, primarily neutrophils. The difference between the observed ROS levels induced by wild-type and Δ*hofQ* mutant *A. actinomycetemcomitans* cells was not significant when bacterial cells were pretreated with plasma from a healthy donor (p = 0.442, Mann-Whitney U-test; (Figure 6(A)). The treatment of bacteria with sera from *A. actinomycetemcomitans*-positive patients tended to enhance the production of ROS by leukocytes compared with that observed when bacteria were treated with healthy control serum (Figure 6(B)), but the change was only significant for the Δ*hofQ* mutant (p = 0,005 Mann-Whitney U-test). In addition, no difference in the induction potential of ROS production was observed between the wild-type and Δ*hofQ* mutant strains when cells were pretreated with either patient serum or healthy control serum (Figure 6(B)).

### The amount of anti-emHofQ antibody was not elevated in the sera of A. actinomycetemcomitans-positive patients

To investigate the possible immunogenicity of emHofQ, we examined whether *A. actinomycetemcomitans*-positive patients have antibodies against emHofQ. EmHofQ was immobilized in microtiter wells, and interactions with human serum antibodies were detected with anti-human antibodies. *A. actinomycetemcomitans*-positive patients exhibited antibody titers against emHofQ that were comparable to those observed with healthy control subjects (p = 0.242, Mann-Whitney U-test, Figure 6(A)). However, the absolute absorbance values were relatively low, and some of the values were close to the background level (Figure 6(C)).

When the specific emHofQ antibody titers from *A. actinomycetemcomitans*-positive patient sera were compared with the induction potential of ROS production of the wild-type and Δ*hofQ* mutant strains, no correlation between the two parameters was observed (r_s_ = 0.503 (wt) and 0.434 (Δ*hofQ*); p = 0.095 (wt) and 0.159 (Δ*hofQ*); *Spearman’s* rank-order correlation).

## Discussion

Our study indicates for the first time that the uptake of host cytokines is associated with the natural competence of bacterial species. This theory is supported by our findings that the host cytokine IL-8 interacted with bacterial DNA and that the secretin channel HofQ, which has earlier been proposed to participate in DNA uptake, interacted with IL-8. Similar results were obtained with IL-1β, with the exception that IL-1β did not bind DNA, and the binding affinity of IL-1β to emHofQ was weaker than that of IL-8. Moreover, both IL-8 and IL-1β decreased the amount of eDNA in the biofilm, and deletion of *hofQ* abolished this effect.

In previous studies, only the interaction between the outer membrane protein Caf1A of *Y. pestis* and IL-1β has been investigated in detail [25]. The binding affinity of that interaction was high, with a K_d_ of 140 ± 14 pM. However, those experiments were performed with intact *E. coli* cells that expressed recombinant Caf1A on their surfaces. While the ability of *A. actinomycetemcomitans* to bind and take up cytokines has been known for some time, emHofQ is the first *A. actinomycetemcomitans* outer membrane protein for which it has been possible to determine the binding constant for a certain cytokine. In addition, although the HofQ homolog NmPilQ has been shown to play a role in IL-8 uptake in *N. meningitides* [19], no binding constants were determined in that study. A specific interaction between emHofQ and IL-8 was detected in both static (K_d_ = 43 nM) and dynamic (K_d_ = 2.4 μM) settings. The binding affinity was approximately 50 times weaker in the dynamic setting than in the static setting, but the dynamic setting better represents the *in vivo* environment of HofQ in the membrane with free diffusion of IL-8. The different functions of proteins reflect their different affinities toward ligands; antibodies bind to their targets very tightly, while receptors exhibit only moderate binding. The interaction between emHofQ and IL-8 cannot be too strong because, presumably, emHofQ functions as a recognition motif that binds to its ligand only temporarily. The interaction was further confirmed by the observation that IL-8 stabilized the structure of emHofQ.

Deletion of *hofQ* abolished the biofilm response to IL-1β and IL-8. The decrease in eDNA levels in response to these cytokines was the most prominent change in the wild-type biofilm, which was consistent with our previous results [17]. Initially, *A. actinomycetemcomitans* HofQ was identified as a DNA-binding protein and a possible transporter of DNA in naturally competent cells [20]. Therefore, the interaction between IL-8 and dsDNA, which we demonstrated in this study, might act as a mechanism that combines cytokine uptake with DNA uptake. Our results are consistent with the earlier finding by Perks and Shute [26], who reported the binding of IL-8 to human placental DNA. Interaction between IL-8 and DNA is likely independent of DNA sequence and is dependent on the physicochemical properties of IL-8 and DNA. In physiological milieus, IL-8 is positively charged, whereas the backbone of DNA has a negative charge; therefore, the opposite charges most likely play a crucial role in the interaction.

Using crosslinking and mutational assays, we identified the interaction site of emHofQ, which binds IL-8: two adjacent loops in the type I KH domain of emHofQ were involved in the binding according to the mutational assays. Although the assay identified the protein-protein interface between emHofQ and IL-8, the mutational assay was not sensitive enough to determine the specific role of any mutated residue. The changes in the single mutants were extensive, involving at least two charged amino acids. Moreover, two of the mutated amino acids, K139 and D165, interacted with each other in wild-type emHofQ, forming a bridge between the two loops. Thus, changing one of these led inevitably to structural changes in the non-mutated loop. Our structural comparisons revealed a homologous domain in emHofQ and NmPilQ at the site where the interaction between emHofQ and IL-8 was indicated to occur. Further mapping of the interaction of cytokines with NmPilQ is needed to establish the point of interaction, but it is tempting to speculate that the type I KH domain fold is recruited for cytokine interaction in both of these bacterial proteins.

In our study, we also demonstrated the binding of IL-1β to emHofQ, but the K_d_ values were lower than those seen with IL-8. Moreover, interaction with emHofQ destabilized the fold of IL-1β, indicating that the interaction unfolds the IL-1β structure, which may facilitate the transfer of the cytokine through the channel. Even though we could not determine the exact interaction sites between emHofQ and IL-1β with the crosslinking method, the mutational assays indicated that the site at which IL-1β binds to emHofQ is most likely the same as that used by emHofQ when interacting with IL-8.

Anti-emHofQ antibody titers were not elevated in the sera of patients who tested positive for *A. actinomycetemcomitans*, which is understandable, because emHofQ domain of the outer membrane secretin channel likely faces the periplasmic space rather than the outside of the cell. The structural homology of this protein to the periplasmic regions of EscC from enteropathogenic *E. coli* [27] and GspD from enterotoxigenic *E. coli* [28] was shown by Tarry and co-workers [20]. Both *A. actinomycetemcomitans* strains used in this study induced the production of ROS by leukocytes, and the induction potential was unaltered after the deletion of *hofQ*, supporting the hypothesis that HofQ is not involved in the recognition process. Treatment of bacteria with sera from *A. actinomycetemcomitans-*positive patients enhanced the production of ROS by leukocytes, which was most likely due to the presence of *A. actinomycetemcomitans-*specific antibodies or opsonization with complement. However, there was no correlation between specific anti-emHofQ antibody levels and the opsonization potential of *A. actinomycetemcomitans*-positive patient serum. Thus, although there are likely some antibodies in the patient sera that are involved in the induction of ROS production by *A. actinomycetemcomitans*, those antibodies are not specific to HofQ.

Deletion of *hofQ* decreased the biofilm formation of *A. actinomycetemcomitans*. Total biofilm mass as well as amounts of all the major EM components, namely, PGA, protein, and eDNA, were diminished. Moreover, deletion of *hofQ* made *A. actinomycetemcomitans* more susceptible to the tested β-lactams (ampicillin and amoxicillin/clavulanic acid) but did not alter the response to tetracyclines. To affect their bacterial targets, antimicrobials need to penetrate bacterial cell walls. Many antimicrobials, including β-lactams [29] and tetracyclines [30], enter bacteria through Omp-like porin channels. β-Lactams break down the cell walls by targeting the penicillin-binding protein in the periplasmic space [31], and tetracyclines, on the other hand, inhibit protein synthesis by binding to the ribosome [32]. However, bacterial cell walls are not the only barrier that antimicrobials need to overcome in bacterial biofilms. The EM can bind various antimicrobials with its versatile components. For example, eDNA interacts with aminoglycosides, thereby protecting *P. aeruginosa* biofilm-associated cells [33]; sub-inhibitory concentrations of penicillin augment the production of all EM components of *Actinobacillus pleuropneumoniae* [34]; and the treatment of *Haemophilus influenzae* biofilms with DNase I makes the cells more sensitive to ampicillin and ciprofloxacin [35]. Deletion of *hofQ* decreased the amount of eDNA while increasing the susceptibility to the tested β-lactams, supporting the hypothesis that eDNA can sequester and protect biofilm-associated cells from the activity of β-lactams. Although the *A. actinomycetemcomitans* Δ*hofQ* mutants were more susceptible to the tested β-lactams than the wild-type strain, the deletion of *pilQ* in *N. gonorrhoeae* made the strain more resistant to penicillin and decreased the transformation efficiency of the strain [36]. However, the *N. gonorrhoeae* strain used harbored determinant of penicillin resistance, *mtrR* and *penB*, which decrease antimicrobial permeation through outer membrane porins.

Bacterial proteins that interact with molecules associated with human immune responses may be more abundant than previously thought. To survive in hostile environments, bacteria must have multiple ways to cope, and in host environments, bacteria could benefit by taking advantage of the host inflammatory mediators [19,37–40]. Moreover, by sequestering host cytokines, bacteria could manipulate the immune signaling pathways of the host, possibly establishing an imbalance that weakens the defense of the host against invaders. This study is the first time that bacterial cytokine binding has been linked to the natural competence of bacteria and uptake of eDNA. Moreover, we were able to determine the binding constants between bacterial proteins and host cytokines as well as characterize the host cytokine interaction site in the bacterial protein for the first time. This type of data is needed in further studies on the role of cytokine binding in bacterial virulence. Comprehensive information regarding protein-protein interaction sites is required in order to specifically perturb the interaction without deleting the whole protein. Many bacterial virulence-associated proteins are moonlighting proteins, *i.e*., these proteins have other tasks, many of which are associated with essential cell functions (for a review, see ref. [41]). Thus, methods to specifically perturb virulence-related interfaces are essential to investigate the functions of these proteins in disease development.

In healthy junctional epithelium, chemokine IL-8 forms a chemotactic gradient that enhances the host defense by facilitating neutrophil migration and phagocytosis. However, in periodontitis, this IL-8 gradient fades, debilitating the host response to potential pathogens [42,43]. In naturally competent *A. actinomycetemcomitans* strains capable of boosting IL-8 sequestration by linking it to DNA uptake, this process may have a role in weakening the host defense in a manner similar to that described above.

## Materials and methods

### Ethics statement

Permission to collect and use blood samples of *A. actinomycetemcomitans*-positive periodontitis patients as well as those of healthy control subjects specifically for this study was obtained from the Ethics Committee of the Hospital District of Southwest Finland. Written informed consent was obtained from *A. actinomycetemcomitans*-positive adult periodontitis patients (12) and healthy controls (12) to collect venous blood samples.

### Bacterial strains, plasmids and growth conditions

The *A. actinomycetemcomitans* D7S wild-type strain (a kind gift from Professor Casey Chen, University of Southern California, Los Angeles, USA) and the mutant strains D7S Δ*hofQ*::Spe and D7S Δ*hofQ* were used in this study. The pLox2-spe and pAT-Cre plasmids [23] (a gift from Professor Casey Chen) were amplified in the *E. coli* strain TOP10 (Invitrogen) by standard methods, and recombinant proteins were produced in the *E. coli* BL21 CodonPlus (DE3) RIL protein expression strain (Stratagene). Unless otherwise stated, *A. actinomycetemcomitans* D7S biofilm growth was performed as described earlier [17], and *A. actinomycetemcomitans* D7S was always grown in a candle jar at 37°C.

### Cloning and expression of recombinant proteins

The production and purification of recombinant emHofQ (without a His tail) and IL-8 has been described by Tarry and co-workers [20] and Ahlstrand and co-workers [17], respectively. To produce C-His emHofQ, the emHofQ sequence (corresponding to *hofQ* residues 27–195) was cleaved from the pET15b plasmid [20] with *Xho*I and *Nde*I and cloned into pET36b (C-terminal His tag, Novagen). Genes encoding the emHofQ mutants were ordered as codon-optimized genes from Eurofins Genomics (Ebersberg, Germany) (Table 3). These genes contained following mutations: H136A, F137A and K139A (emHofQ_L1); D165A and R166A (emHofQ_L2); or all five (emHofQ_L1L2). The genes were cloned into the pET36b vector in order to produce proteins with C-terminal His-tags. The codon-optimized gene of human mature IL-1β (amino acids 117–269) was ordered from Eurofins Genomics (Table 3), amplified in the pET15b vector (Novagen), and moved into the pET-28b-based pET-28bTEV plasmid, which contains an N-terminal His tag with a tobacco etch virus (TEV) protease cleavage site instead of a thrombin digestion site.

Verified expression constructs were transformed into the *E. coli* BL21 CodonPlus (DE3)RIL protein expression strain. The proteins were expressed in Terrific broth (12 g/l tryptone, 24 g/l yeast extract, 0.4% glycerol, 17 mM KH_2_PO_4_, and 72 mM K_2_HPO_4_) supplemented with 30 μg/ml kanamycin and 30 μg/ml chloramphenicol. Expression was induced with 1 mM isopropyl β-D-1-thiogalactopyranoside (IPTG) when the OD_600nm_ reached 1.2. Cells were harvested by centrifugation (7000 × g, 10 min, 4°C) after growth for 2.5 h. When producing emHofQ mutants, expression was induced at an OD_600nm_ of 0.8, and cultures were grown overnight at 16°C before harvesting. All cell pellets were stored at −20°C. Purification was performed from 5 g of thawed cells that were suspended in 30 ml of buffer A (20 mM NaH_2_PO_4_/Na_2_HPO_4_, 300 mM NaCl, and 20 mM imidazole, pH 7.5) supplemented with DNase I (Roche, #10,104,159,001), 0.2 mM phenylmethylsulfonyl fluoride (PMSF; Sigma, P-7626) and 10 mM MgCl_2_. The cell suspension was sonicated 5 times for 15 s each time with a 100-watt MSE ultrasonic disintegrator (amplitude, 10 μm) and centrifuged for 48 000 × g for 30 min at 4°C to remove the cell debris. The supernatant containing overexpressed protein was loaded onto a 5-ml HisTrap HP column (GE Healthcare, 17–5248-01); the column was washed with buffer A; and the protein was eluted with buffer A supplemented with 250 mM imidazole. The eluted proteins were further purified by size-exclusion chromatography on a Superdex 200 26/60 column (GE Healthcare, 28–9893-36) that was equilibrated with PBS. The N-terminal 6× His tag of IL-1β was removed by digestion with TEV protease before size-exclusion chromatography. One milligram of recombinant TEV protease supplemented with 1 mM DTT and 0.5 mM EDTA was used per 20 mg of IL-1β, and the overnight incubation was carried out at RT. Cleaved IL-1β was collected from the flow through from the HisTrap column.

Fractions containing proteins of the correct size were pooled and concentrated with 10-kDa an Amicon® Ultra-15 centrifugal filter device (Merck Millipore, UFC901008). SDS-PAGE was used to verify protein purity, and the protein aliquots were stored at −80°C.

### ELISA-based cytokine-binding assay

Each well of a 96-well plate (Nunc Maxisorp, Thermo Fisher Scientific, #442,404) was coated with 6 pmol of cytokines diluted in PBSN (PBS with 0.05% sodium azide), and the plate was incubated at RT overnight. The cytokines used were IL-1β (production described above), IL-8 [17], IL-6 (#200–031), TNF-α (#300–028) and IFN-γ (#100–039) (ReliaTech GmbH). Each reaction was performed in triplicate, and BSA was used as a negative control. The following day, the wells were washed three times with ion-exchanged water, blocked with 0.25% BSA in PBS-T (0.05% Tween-20 in PBS) for 3 h at 37°C, and washed as above. Next, 490 nM emHofQ with a C-terminal 8-His tail in PBS was incubated at 4°C overnight, which was followed by three washes with PBS-T using Delfia Platewash (Perkin Elmer). Fifty microliters of 1/5000 dilutions of His-Probe-HRP™ (Thermo Fisher Scientific, #15,165) in PBS-T were incubated in the wells at RT for 15 min. Wells were washed with PBS-T as described above and treated with 2.2´-azino-bis(3-ethylbenzothiazoline-6-sulfonic acid) diammonium salt (Sigma-Aldrich, A9941) in citrate buffer (10 mM sodium citrate and 0.03% H_2_O_2_, pH 4.2) for detection. Absorbance was measured at 405 nm using a Multiskan GO plate reader (Thermo Fisher Scientific). The obtained values were compared with those of the negative control, and the results from four independent experiments were combined.

### Time-resolved fluorometric immunoassay (TRFIA)

Wells of a Nunc Maxisorp 96-well plate (Thermo Fisher Scientific) were coated with 6 pmol of IL-1β or IL-8 in PBS at 4°C overnight. BSA was used as a negative control. Unbound proteins were washed away with PBS using Delfia Platewash (Perkin Elmer). Empty binding sites were blocked with 200 μl of Alternative Block (BB5, ImmunoChemistry Technologies, #6299) at RT overnight, after which the wells were washed 3 times as described above. Serial dilutions of C-His-emHofQ (from 10 to 740 nM) in Delfia Assay Buffer were incubated in wells at RT for 1 h, after which the plate was washed as described above. Twenty-five nanograms of Delfia Eu-N1 anti-6xHis antibody (Perkin Elmer, AD0108) in 50 μl of Delfia® Assay Buffer was incubated in wells at RT for 1 h, after which the wells were washed as described above. A 5-min incubation with DELFIA® Enhancement Solution (Perkin Elmer, 4001–0010) was followed by measuring time-resolved fluorescence using a Victor [3] multilabel plate reader (Perkin Elmer). Each sample was prepared in triplicate, and three independent experiments were performed.

TRFIA with the emHofQ mutants was performed by using the same protocol, with the exception that wells were coated with 60 pmol of IL-8, IL-1β or BSA, the concentrations of emHofQ mutants used ranged from 5 nM to 2 μM, and the emHofQ dilutions were incubated at 4°C overnight.

### Binding of IL-8 and IL-1β to emHofQ examined by SPR

Binding of IL-8 to emHofQ was studied by SPR (Biacore X, GE Healthcare). An NTA Chip (GE Healthcare, BR-1000–34) was activated with a 2-min pulse of 500 μM NiCl_2_ in elution buffer (10 mM HEPES, 150 mM NaCl, and 0.005% P-20 surfactant, pH 7.4) using a flow rate of 5 μl/min. After activation, N-His-emHofQ was coupled to the surface (4 μl of 10 nM emHofQ in the elution buffer), resulting in 115–199 RU of bound protein. At this concentration of emHofQ, the coupling led to a stable baseline. The binding of IL-8 was studied by monitoring the response of 60 μl of protein injected at concentrations of 1–15 000 nM at a flow rate of 30 μl/min. An uncoupled surface was used as a reference, and the readings from a buffer injection were subtracted from those for each protein injection. After each injection, the surface was regenerated with a 10-μl pulse of 350 mM EDTA in the elution buffer followed by 2 μl of 0.2 M NaOH. A new emHofQ surface was prepared for each IL-8 concentration. The binding constant for the interaction between IL-8 and emHofQ was determined by plotting the binding (RU) at the peak or at the end of the injection (as observed at the higher concentrations, the equilibrium was not reached, presumably due to nonspecific binding) as a function of IL-8 concentration by using a one-site binding model in Origin (OriginLab). The apparent binding constant was calculated as an average of three independent measurement series.

The binding of recombinant IL-1β to immobilized emHofQ was studied almost identically as that of IL-8. For this experiment, 3 μl of 10 nM emHofQ was used for coupling, and IL-1β concentrations from 1 nM up to 50 μM were used. The binding constant was not determined due to moderate binding.

### Thermal shift assay

To determine the effect of the interaction on the thermostability of emHofQ, the concentration of emHofQ was kept constant at 5 μM, whereas the concentration of IL-8 was varied from 5 to 200 μM. When the interaction between emHofQ and IL-1β was studied, the IL-1β concentration was kept constant at 5 μM, and the emHofQ concentration was varied from 2.5 to 50 μM. Duplicate reactions were performed in PBS supplemented with 2.5 μl of 0.4% SYPRO orange (Thermo Fisher Scientific, S6650) in a total volume of 25 μl in a white multiwell PCR plate (Bio-Rad). The plate was sealed with Microseal® B adhesive sealer (Bio-Rad), and the reagents were mixed by centrifugation at 1000 rpm for 1 min. Differential scanning fluorometry was performed with a MiniOpticon real-time PCR system (Bio-Rad). Briefly, the plate was heated from 20 to 99°C, and the fluorescence was monitored in the range of 540–700 nm every 0.5°C. T_m_ values were estimated from the fluorescence curves using Opticon Monitor 3 software. The T_m_ values obtained from six (IL-8) or four (IL-1β) independent experiments were combined.

### Chemical crosslinking, in-gel digestion and nano-LC/MS/MS analysis of crosslinked peptides

To identify the interaction sites, emHofQ and IL-8 were incubated separately and together, with and without the crosslinking agent. The reaction was prepared in conjugation buffer (100 mM sodium phosphate buffer, 0.15 M NaCl, pH 7.4) in a total volume of 10 μl, containing 5 μM studied protein(s) and 3 mM crosslinking agent bissulfosuccinimidyl suberate (BS3, in MQ). The reaction mixture was incubated for 30 min at RT, and the reaction was stopped by adding 1 M Tris-HCl (pH 8.5) at a final concentration of 50 mM. The incubation was continued for 15 min, and the samples were subsequently stored at −20°C. Samples were electrophoresed on a Criterion 4–15% Tris-HCl gel (Bio-Rad, 345–0028) and silver stained [44]. Bands that were the correct size and appeared only when emHofQ was incubated with IL-8 in the presence of the crosslinker were excised. The excised gel bands were destained in 1:1 acetonitrile (ACN):100 mM ammonium bicarbonate with vortexing, reduced with 10 mM dithiothreitol at 56°C, and alkylated with 50 mM iodoacetamide. Overnight in-gel digestion was carried out with 10 ng/µl dimethylated porcine trypsin (Sigma-Aldrich) at 37°C. Peptides were extracted using bath sonication followed by 30 min of vortexing in 2 volumes of 1:2 5% formic acid (FA):ACN. The organic phase was evaporated in a vacuum centrifuge, and the peptide mixture was desalted with a homemade C18 (3M Empore) solid-phase extraction tip.

The digested samples were injected into an Ultimate 3000 RSLCnano liquid chromatography system (Dionex/Thermo Fisher Scientific) using a C18 trap column (Dionex) and a self-packed (3-µm C18 particles, Dr. Maisch) analytical 50 cm× 75 µm emitter column (New Objective). Peptides were eluted at 200 nl/min with an 8–40% B 90-min gradient (buffer B: 80% ACN + 0.1% FA; buffer A: 0.1% FA) into a Q Exactive Plus (Thermo Fisher Scientific) tandem mass spectrometer operating with a top-10 data-dependent acquisition strategy. Briefly, one 400–1600 m/z MS scan at a resolution setting of R = 70,000 (at 200 m/z) was followed by higher-energy collisional dissociation fragmentation (normalized collision energy of 27) of the 10 most intense ions (charge states: + 3 to + 6) at R = 35,000. MS and MS/MS ion target values were 3e6 and 5e4 with injection times of 50 and 120 ms, respectively. Dynamic exclusion was limited to 25 s.

### Identification of crosslinked peptides from raw mass spectrometric data

Proteins in the MS sample were first identified with the MaxQuant Andromeda search engine [45] using the UniProt (www.uniprot.org) *E. coli* K12 reference proteome database supplemented with human IL-8 and HofQ sequences. Proteins with at least 2 identified peptides along with human IL-8 and HofQ sequences were included into the crosslinked peptide search along with the reversed-sequence counterparts. The search was performed with SIM-XL software 1.2.0.3 [46]. BS3 was defined as the crosslinking agent, and carbamidomethylation of cysteine and oxidation of methionine were defined as the fixed and variable modifications, respectively. Trypsin specificity (cleavage after K and R) was defined as the cleavage rule with a maximum of three miscleavages allowed. Precursor and fragment ion tolerances were set to 5 and 20 ppm, respectively. Search filter parameter thresholds were set such that no proteins were identified based on the reverse sequences. Additionally, at least 5 peaks per chain were required for identification. This resulted in 16 and 30 identified crosslinked peptide species per monomer and oligomer band, respectively, and an estimated false discovery rate (FDR) of ≤ 5%.

### Binding of dsDNA to IL-8 examined by EMSA

IL-8 binding to the linearized USS plasmid (dsDNA) was examined by a method described by Tarry and co-workers [20]. Briefly, 31 μg/ml of linearized USS plasmid was incubated with recombinant IL-8 (ranging from 20 ng/ml to 100 μg/ml) in PBS at RT for 30 min. BSA (10 mg/ml) was used a negative control. Samples were loaded into a 0.8% agarose gel and run at 70 V for 2 h. Bands were visualized using Midori Green Advance stain (Nippon Genetics Europe, MG04) under a blue LED light.

### Site-specific deletion of the hofQ gene

**Figure 7.**
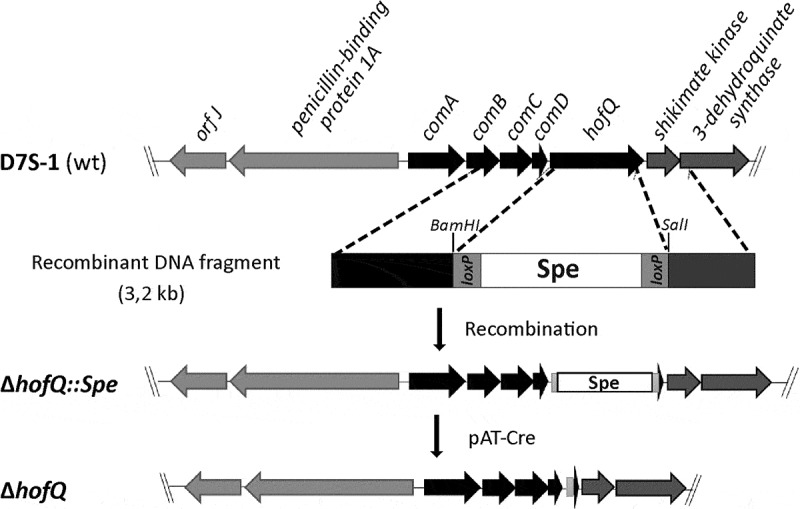
Preparation of the markerless D7S Δ*hofQ* mutant. Natural transformation and homologous recombination was used to replace *hofQ* with the recombinant *loxP-spe-loxP* cassette that contained the flanking regions upstream and downstream of *hofQ*. The transformants (Δ*hofQ::spe*) were verified by PCR. The *loxP-spe-loxP* cassette was removed by the pAT-Cre-plasmid (Tet^R^), which was introduced via natural transformation. Cre recombinase then catalyzed the recombination between the *loxP* sites. The obtained colonies were examined for Spe and Tet resistance, and the colonies sensitive to both antimicrobials had lost both the *loxP-spe-loxP* cassette and the pAT-Cre plasmid, *i.e*., these colonies were markerless D7S Δ*hofQ* mutants.

The construction of the single gene deletion mutant D7S Δ*hofQ* was performed using the site-specific Cre*/loxP* gene deletion system as previously described (Figure 7) [17,23,24]. Primers and annealing temperatures used for target gene deletion and genomic mutant quantification are listed in Table 4. In brief, an 8.2-kb PCR product containing the *hofQ* gene (NC_017846.2, region 1,045,184.1046593) was amplified from the genome of *A. actinomycetemcomitans* D7S using the *hofQ*_nest primers. Next, the upstream region of *hofQ* was amplified using the primers comB_F and HofQ_R_BamHI, and the downstream region was amplified using the primers HofQ_F_SalI and 3DHQ synthase_R. PCR fragments and the pLox2-spe plasmid were digested with *Bam*HI and/or *Sal*I restriction enzymes (FastDigest restriction enzymes, Thermo Fisher Scientific), which was followed by ligation (approximately 130 ng of each fragment) of the *lox2-spe* cassette between the upstream and downstream regions of *hofQ* with T4 DNA ligase (Thermo Fisher Scientific, #EL0014). The resulting recombinant PCR product (3.2 kb) was used for natural transformation of *A. actinomycetemcomitans* D7S. Colony PCR analysis of spectinomycin (*spe*)-resistant colonies indicated that the *hofQ* gene had been replaced by the *spe* cassette. The *spe* cassette was identified using the Spe_Urf/Spe_DWR2 primer pair for PCR. In addition, colony PCR with the nest primers followed by *Sal*I digestion of the amplification product was used to verify the correct primary mutation in D7S Δ*hofQ::spe*. The genomic *spe* cassette was deleted using the Cre/*loxP* recombination system [23,24]. First, the pAT-Cre plasmid was transformed into the D7S Δ*hofQ::spe* mutants using electroporation. The potential markerless D7S Δ*hofQ* mutants were first cultured on a plate supplemented with tetracycline and then on antimicrobial-free plates. Colony PCR with the Spe_primer pair was used to verify the removal of the *spe* cassette. Finally, genomic DNA was isolated using a QIAamp® DNA Mini Kit (Qiagen, #51,304), and the flanking regions of the *hofQ* gene were amplified from genomic DNA using the HofQ_seq primer pair. The product was sequenced in both directions by Eurofins Genomics using the primer pair HofQ_F_seq2/3DHQ_R2_seq2 to verify the absence of *hofQ* in the genome of the D7S Δ*hofQ* strain.

**Table 1.** Formation and composition of *A. actinomycetemcomitans* D7S Δ*hofQ* biofilm compared to that of the wild-type (wt) D7S strain.

	Biofilm mass	eDNA	PGA	Total protein
% of wt	45 ± 22	57 ± 12	78 ± 9	54 ± 13
(p-value)	(0.029)	(0.029)	(0.029)	(0.029)

**Table 2.** Effect of *hofQ* deletion on the biofilm response to cytokines IL-1β and IL-8, measured as changes in biofilm composition.

		Percentage of the control (without cytokines)			
Strain	Cytokine	Biofilm mass	eDNA	PGA	Total protein
**D7S wt**	IL-1β	103 ± 7	70 ± 14	105 ± 5	85 ± 11
p-values			(0.029)	(0.029)	(0.029)
**D7S wt**	IL-8	96 ± 2	65 ± 18	88 ± 19	90 ± 20
p-values		(0.029)	(0.029)		
**D7S *ΔhofQ***	IL-1β	95 ± 15	116 ± 29	98 ± 5	102 ± 8
**D7S *ΔhofQ***	IL-8	89 ± 15	113 ± 39	97 ± 7	100 ± 29

**Table 3.** Codon-optimized genes for protein expression. Restriction enzyme binding sites (*Nde*I and *Xho*I) are underlined.

Gene name	Codon-optimized sequence
*mature_il-1β*	CAT ATG GCC CCT GTA CGT TCC CTC AAT TGC ACC TTA CGC GAT AGT CAG CAG AAA TCT TTG GTG ATG TCA GGT CCC TAC GAA CTG AAA GCA CTC CAT CTG CAA GGA CAG GAT ATG GAA CAG CAA GTC GTG TTT AGC ATG AGC TTT GTA CAA GGC GAA GAA TCG AAC GAC AAA ATT CCA GTT GCT CTT GGC CTG AAA GAA AAG AAC CTG TAT CTG TCG TGT GTC TTG AAA GAT GAT AAA CCG ACA CTG CAG TTA GAA AGT GTT GAT CCG AAG AAT TAT CCG AAG AAG AAA ATG GAG AAA CGC TTT GTG TTC AAC AAA ATC GAG ATC AAC AAC AAA CTG GAG TTC GAA TCT GCG CAA TTT CCG AAT TGG TAC ATT AGC ACC TCA CAG GCG GAA AAT ATG CCA GTG TTT CTG GGT GGG ACT AAA GGC GGT CAG GAC ATT ACG GAC TTC ACC ATG CAG TTC GTT TCC AGC TAA CTC GAG
*emhofQ_L1*	CAT ATG CAG AAT CCG GTG TTC TCG ATT CGC TTG AAA CAA GCG CCA CTC GTA CCG ACT TTA CAG CAG TTG GCG TTA GCG CAT AAC ACC AAC CTG ATT ATC GAC GAT GAA CTG CAA GGT ACG GTG AGT CTG CAA CTG GAA AAC GTG GAT CTG GAC CAG CTC TTT CGT AGC GTT GCG AAG ATT AAG CAG CTG GAT CTG TGG CAG GAG AAT GGC ATC TAT TAC TTC ACC AAA GGC GAT ACC AAC ACG AAA TTT GCG GGG AAA ATG GAG GAA CCG TTT CCG TTA TCT CTG CCT ATG GCC GAA GAA CCC GCT CAG CTT AAC ACA GCC ACC ATC AAA CTT GCA GCT GCA GCA GCC TCT GAA GTC ATG AAA TCG CTC ACT GGT GGT TCA GGA AGC CTG CTT TCC CCA AAT GGC TCC ATT ACG TTC GAT GAC CGC AGC AAT TTG CTG CTG ATT CAG GAT GAA CCT CGT AGT GTT CGC AAC ATC AAG AAA CTG ATC AAA GAG CTG GAC AAA CCG ATT GAA CAA CTC GAG
*emhofQ_L2*	CAT ATG CAG AAT CCG GTG TTC TCC ATT CGT CTG AAA CAA GCA CCT CTC GTT CCG ACA CTT CAG CAG TTA GCG TTA GCC CAT AAC ACC AAC CTG ATT ATT GAC GAT GAA CTC CAA GGC ACG GTT AGC CTG CAA CTG GAG AAT GTG GAC TTG GAT CAG CTG TTT CGC AGT GTA GCG AAA ATC AAA CAG CTG GAT CTT TGG CAG GAA AAC GGG ATC TAC TAT TTC ACC AAA GGT GAT ACG AAC ACC AAA TTT GCG GGC AAA ATG GAG GAA CCG TTT CCG CTT AGC CTG CCA ATG GCA GAA GAA CCA GCT CAG TTG AAT ACT GCG ACG ATC AAA CTG CAC TTT GCC AAA GCT TCC GAA GTG ATG AAG TCG TTG ACT GGT GGT AGC GGA TCT CTG CTC TCA CCG AAT GGC TCG ATT ACC TTC GAT GCA GCC TCT AAC CTG TTA CTG ATT CAA GAC GAA CCT CGT AGT GTC CGC AAC ATC AAG AAG CTG ATC AAA GAG CTG GAT AAA CCC ATT GAA CAG CTC GAG
*emhofQ_L1L2*	CAT ATG CAG AAT CCC GTT TTC AGC ATT CGC CTG AAA CAA GCG CCT TTG GTA CCG ACC CTT CAG CAG TTA GCC TTG GCG CAT AAC ACC AAC CTG ATT ATT GAC GAT GAA CTG CAA GGT ACT GTG TCT CTG CAG TTA GAG AAT GTG GAT CTG GAC CAG TTG TTC CGT TCA GTC GCG AAA ATC AAA CAG CTT GAT CTG TGG CAA GAG AAC GGC ATC TAC TAT TTC ACG AAG GGT GAT ACC AAC ACC AAA TTT GCC GGG AAA ATG GAA GAA CCG TTT CCG CTG TCG TTA CCT ATG GCT GAA GAA CCA GCG CAA CTC AAC ACA GCG ACG ATC AAA CTG GCT GCT GCA GCC GCA AGT GAA GTG ATG AAA TCC CTG ACT GGA GGT AGT GGC TCT CTG CTG TCC CCG AAT GGC TCG ATT ACG TTT GAT GCC GCA AGC AAT CTT CTC CTG ATT CAG GAT GAA CCA CGT AGC GTT CGC AAC ATC AAG AAG CTC ATC AAA GAG CTG GAC AAA CCG ATT GAA CAG CTC GAG

**Table 4.** Primers and annealing temperatures used in this study. Restriction enzyme sites are underlined.

D7S Δ*hofQ* generation		
Primer name (code)	Sequence 5`→ 3`	T_m_ annealing (°C)
HofQ_nest_F (01)	TTGGTTCCGTTCCTTTAATGAAATATTC	70.0
HofQ_nest_R (02)	ATAGCATGACCAAAGGTATGACCGAGA	
ComB_F (03)	TACAGCCACAGTCAAGAAGAA	60.6
HofQ_R_BamHI (04)	TATGGATCCTGCTATTTCCTTCAAAAGTTC	
HofQ_F_SalI (05)	TAAGTCGACCTAAATAGGAAATAA	46.2
3DHQ synthase_R (06)	GTAGCTTCTTTCTTTCAA	
Spe_Urf (07)	GCCACTGCATTTCCCGCATA	69.1
Spe_DWR2 (08)	TGCAGGTCGATTTTCGTTCGT	
HofQ_seq_F1 (09)	ATGTTACACCCTCTTTTC	51.2
HofQ_seq_R1 (10)	TGCCATTGTTATAATCTTA	
HofQ_F1_seq2	ATTCTATTCACAGCGAAC	*
3DHQ_R2_seq2	TTCAGCGGATAACAATG	
**Reverse transcription PCR**		
16 S_F	TACGCATTTCACCGCTACA	59.1
16 S _R	ATGCCAACTTGACGTTAAAT	
HofQ_F_rt1 (13)	TCTTATCGTCAGCCAAAACTCG	63.9
HofQ_R_rt1 (14)”	TATCGCTAAATACACCGCCC	
ClpX_F (15)	GGTTATGTGGGCGAAGATG	64.0
ClpX_R (16)	CGATTTGCGGGTGATTTTG	
ComA_F (17)	TTGCCCCATAACCTGAAC	57.8
ShiKi_I_R (18)	GTAGATAACACGATGCCTT	
ComD_O_F (19)	TTTGCGTTGGGATAACCC	63.7
HofQ_O_R (20)	ACAGACCGCACTTTAGCCAT	

*performed by Eurofins Genomics

”Annealing temperature of 61.5°C with primer pair 14/17

We attempted to restore the expression of HofQ in the markerless Δ*hofQ* mutant using an *A. actinomycetemcomitans*/*E. coli* shuttle plasmid that contains a constitutively expressed leukotoxin promoter, as described previously [17]. However, the strains were non-viable, likely due to deleterious changes in the outer membrane stability.

### RNA extraction and analysis

*A. actinomycetemcomitans* D7S and D7S Δ*hofQ* cells (1 × 10^9^ CFU) were cultured in modified TSB (mTSB) medium (3% tryptone soy broth supplemented with 0.8% glucose) in 50-ml cell culture bottles (Cellstar #690,160, Greiner Bio-One) for 24 h. Half of the biofilm cultures were collected directly, and the other half were washed once with RPMI 1640 medium (Sigma-Aldrich, R7509) supplemented with 0.6 g/l L-glutamine (Sigma-Aldrich, G7513)] before culturing for 2 h in RPMI solution. Biofilm samples were collected and stored in RNAlater® (Invitrogen, AM7020) at 4°C. Before RNA isolation, the RNAlater solution was removed, and the biofilms were washed once with RNAse-free water (Ambion, AM9938) at 6000 × g at 4°C for 5 min. RNA was extracted using a RiboPure™-Bacteria Kit (Ambion, AM1925) according to the manufacturer’s instructions with the following modifications. After resuspending the bacterial cells in RNAwiz solution, the cells were de-clumped by 10 passages through an 18-gauge needle. DNA was removed from the samples by DNase I treatment. PCR amplification of the 16S rRNA gene with 16S primers was performed to confirm that the purified RNA samples were free from DNA contamination [47].

***Reverse transcription PCR*** was used to confirm the Δ*hofQ* deletion at the transcriptional level and to study *hofQ* expression in two different culture conditions. In addition, cDNA was used in PCR analysis to determine whether *comA-hofQ* was transcribed as an operon, as predicted by the Database of prOkaryotic OpeRons [48]. The primers and annealing temperatures used for amplification are indicated in Table 4. Primer pairs comA_F (17)/hofQ_R_rt1 (14), comA_F (17)/shiKi_I_R (18) or comD_O_F (19)/hofQ_O_R (20) were used to further study the organization of the putative operon. Equal amounts of DNase I-treated RNA (1 µg) were used to synthesize cDNA according to the manufacturer’s protocol for the SuperScript® VILO™ cDNA Kit (Thermo Fisher Scientific, #11,754,050). The expression of *hofQ* and that of the reference gene *clpX* (ATP-dependent Clp protease ATP-binding subunit ClpX) was determined by standard PCR (Phusion™ high-fidelity DNA polymerase, Thermo Fisher Scientific, F530L). ClpX was used as a control gene since this gene is known to be expressed in *A. actinomycetemcomitans* biofilm cultures [49]. PCR was performed using 100 ng of cDNA as a substrate. Genomic DNA was used as a template for the positive control, and no template was used for the negative control. For visualization, the amplicons were analyzed on agarose gels stained with Midori Green Advance DNA stain.

### Effect of cytokines on the formation of wild-type and ΔhofQ mutant biofilms

The role of HofQ in biofilm formation was studied with a crystal violet staining procedure [50] as described earlier [17]. Briefly, a 48-well microtiter plate was inoculated with diluted starter cultures of the *A. actinomycetemcomitans* D7S or D7S Δ*hofQ* strains so that each well contained 3.8 × 10^7^ CFU. Four replicates were prepared in each experiment. After being grown for 5 h in mTSB, the biofilms were washed briefly with RPMI solution, and the cultivation was continued in RPMI solution supplemented with 10 ng/ml cytokine (IL-8 or IL-1β). Control cultures were performed without cytokines. After 20 h, culture media were replaced with fresh media, and culturing was continued for 5 h. Finally, the biofilms were stained with crystal violet, and the amount of biofilm formed was quantified by measuring the A_620nm_ values with a Multiskan GO plate reader (Thermo Fisher Scientific). Cytokine-treated biofilms were compared with the equivalent control, and the results from four independent experiments were combined.

### Effect of cytokines on the composition of wild-type and ΔhofQ mutant biofilms

The amount of extracellular PGA in the biofilm cultures was estimated using a Congo-red-binding assay [11] as described earlier by Ahlstrand and co-workers [17]. Biofilm cultures for this assay were prepared in 48-well microtiter plates as described above.

Biofilm cultures for the total protein assay and eDNA extraction were initiated by inoculating 50-ml cell culture bottles (Greiner Bio-One, #391–3103) with 1 × 10^9^ CFU of *A. actinomycetemcomitans* D7S or D7S Δ*hofQ* strains suspended in mTSB medium. After 5 h of pre-culturing, the biofilms were washed once with RPMI 1640 solution, and culturing was continued for 15 h in RPMI solution supplemented with 10 ng/ml IL-8 or IL-1β or in the absence of cytokines. On the following day, the media were changed to fresh media, and culturing was continued for 5 h. Finally, the biofilms were washed three times with PBS (2.7 mM KCl, 1.8 mM KH_2_PO_4_, 140 mM NaCl, and 10 mM Na_2_HPO_4_, pH 7.4), collected by scraping, and suspended in 2.5 ml of PBS. The samples from each culture bottle were divided into two 1.25-ml aliquots, and the biofilm mass of each sample was determined as the weight of the centrifuged pellet. The protocols for the measurement of total protein and eDNA levels in biofilm pellets has been described by Ahlstrand and co-workers [17]. Four technical replicates were performed in each experiment. The results from the cytokine-treated biofilms were compared with those from the equivalent controls, and the results from four independent experiments were combined.

### Antimicrobial susceptibility test

The *A. actinomycetemcomitans* D7S wild-type and *ΔhofQ* mutant strains were grown for 3 days in TSA-blood plates in candle jars at 37°C. Next, 200 µl of bacterial suspension prepared in PBS, equal to 1 McFarland unit, was plated onto *Haemophilus* test medium agar plates (Beckton Dickinson, #254,058). Each plate was supplemented with one Etest® strip (ampicillin, amoxicillin/clavulanic acid, tetracycline or doxycycline, BioMérieux). The plates were incubated at 35°C for 45–48 h before measuring the minimum inhibitory concentration (MIC). The CLSI antimicrobial susceptibility testing breakpoint table (M100, 2017, table for *H. influenzae*) was utilized to interpret MIC values. Mean MIC values from 5–9 independent experiments were reported.

### Collection of human sera

Human venous blood was collected from *A. actinomycetemcomitans*-positive adult periodontitis patients (12) and healthy controls (12), with written informed consent, at the Community Dental Health Care Center of Turku (Institute of Dentistry, University of Turku) by a laboratory nurse or at the Unit for Specialized Oral Care in the Helsinki Metropolitan Area and Kirkkonummi (Helsinki, Finland) by a physician. The patient samples were collected at the beginning of periodontal treatment. *A. actinomycetemcomitans* was detected in the subgingival biofilm samples via either PCR or culturing. Both chronic and aggressive periodontitis patients were included in the study. Patients were excluded if they had been treated with antimicrobials during the previous three months, were pregnant, had severe health problems or were on immunosuppressive medications. Patient smoking behavior was recorded.

### ROS production by leukocytes

The induction of ROS production in leukocytes caused by the *A. actinomycetemcomitans* D7S wild-type and *ΔhofQ* mutant cells was studied using a luminol-amplified chemiluminescence experiment [51] with some modifications. Briefly, triplicate reactions were prepared in a white 96-well plate (Greiner Bio-One, #9,502,887) in a total volume of 250 µl: 100 µl of bacterial suspension (1.25 × 10^7^ CFU), 10 µl of luminol (10 mM in borate buffer), 3 µl of human plasma/serum, 50 µl of human leukocytes (1/3 dilution) and 87 µl of gHBSS (1 mg/ml gelatin in HBSS: 1 mM CaCl_2_, 5 mM KCl, 0.4 mM KH_2_PO_4_, 0.5 mM MgCl_2_∙6H_2_O, 0.4 mM MgSO_4_∙7H_2_O, 140 mM NaCl, 4 mM NaHCO_3_, 0.3 mM Na_2_HPO_4_, and 6 mM glucose). A homogenous bacterial suspension was prepared from three-day plate cultures of the *A. actinomycetemcomitans* D7S wild-type or *ΔhofQ* mutant strains. Plasma/serum was collected from 3 ml of (EDTA-coagulated) whole blood after centrifugation (1000 × g, 10 min, RT), and leukocytes were freshly isolated from 1 ml of (EDTA-coagulated) whole blood following a protocol by Lilius and Nuutila [51]. Measurement of chemiluminescence (every 2 min for 200 min; Victor [3] multilabel plate reader (Perkin Elmer) or Hidex Sense microplate reader (Hidex)) was started immediately after leukocytes were added to the reaction. When assessing the induction of ROS production in leukocytes by the *A. actinomycetemcomitans* D7S wild-type and *ΔhofQ* mutant strains, the plasma used was obtained from the same healthy individual, while the leukocytes were from seven different healthy individuals. When the opsonization efficiencies of different sera were studied, the leukocytes used were obtained from one healthy individual. The peak values of each reaction were compared.

### Anti-HofQ antibodies in A. actinomycetemcomitans-positive patient sera examined by ELISA

Recombinant emHofQ (500 ng) or BSA as a control in PBSN were incubated at RT overnight in a Nunc Maxisorp 96-well plate. Wells were washed three times with ion-exchanged water (here as well as between all subsequent steps). Empty binding sites were blocked with blocking buffer (0.25% BSA and 0.05% Tween-20 in PBS) at RT for 30 min. Patient sera (patients with periodontitis and healthy controls) were diluted to 1/100 in blocking buffer; 50 μl of those dilutions were added into the wells; and the plate was incubated at RT overnight. Wells were blocked again with the blocking buffer at RT for 10 min. Fifty microliters of a 1/6000 dilution (in 0.05% Tween-20, PBS) of anti-human IgG (Fc specific) peroxidase-coupled antibody (Sigma-Aldrich, A0170) was incubated at RT overnight. The detection of peroxidase is described above in “ELISA-based cytokine binding assay”. Initial tests contained two sera per plate at the following dilutions: 1/20, 1/100, 1/500, 1/1000 and 1/2500. The 1/100 dilution was chosen for the combination plates, which contained 12 sera per plate. All reactions were performed in triplicate. A total of 12 patient sera and 12 control sera were tested. Nonspecific binding to the negative control (BSA) was subtracted from the data obtained for emHofQ binding.

### Statistics

The effects of cytokines on biofilm components were analyzed by the Kruskal-Wallis test followed by paired Mann-Whitney U-tests (IBM SPPS Statistics 22), where the experiments conducted in the presence of cytokine were compared pairwise to experiments that lacked cytokine supplementation. The Mann-Whitney U-test was also used while evaluating the effect of the *hofQ* deletion on antimicrobial susceptibility and the induction potential of ROS production. Spearman’s rank-order correlation (IBM SPPS Statistics 22) was used to compare the correlation between antibody titers with the opsonization efficacy of human sera.

## Abbreviations

BSA: bovine serum albumin;
cDNA: complementary DNA;
CLSI: Clinical and Laboratory Standards Institute;
DNA: deoxyribonucleic acid;
dsDNA: double-stranded DNA;
eDNA: extracellular DNA in biofilm;
EM: extracellular matrix;
EMSA: electrophoretic mobility shift assay;
EmHofQ: N-terminal extramembranous domain of the secretin HofQ:
KH domain: K homology domain;
His: histidine;
HofQ: outer membrane secretin protein;
IL: interleukin;
IPTG: isopropyl β-D-1-thiogalactopyranoside;
K_d_: dissociation constant;
Lys: lysine;
MIC: minimum inhibitory concentration;
NMR: nuclear magnetic resonance;
NmPilQ: outer membrane secretin protein of *Neisseria meningitidis;*
PDB: protein data bank;
PGA: poly-N-acetylglucosamine;
PMSF: phenylmethylsulfonyl fluoride;
SPR: surface plasmon resonance;
RT: room temperature;
T_m_: melting temperature;
TRFIA: time-resolved fluorometric immunoassay;
USS: uptake signal sequence
